# An implementation framework for the feedback of individual research results and incidental findings in research

**DOI:** 10.1186/1472-6939-15-88

**Published:** 2014-12-23

**Authors:** Adrian Thorogood, Yann Joly, Bartha Maria Knoppers, Tommy Nilsson, Peter Metrakos, Anthoula Lazaris, Ayat Salman

**Affiliations:** Centre of Genomics and Policy, McGill University, 740, avenue Dr. Penfield, suite 5200, Montreal, QC H3A 0G1 Canada; Faculty of Medicine, McGill University, Montreal, Canada; Research Institute of the McGill University Health Centre, Montreal, Canada; General Surgery, McGill University Health Centre, Montreal, Canada

**Keywords:** Genetics, Genomics, Research ethics, Incidental findings, Individual research results, Participant feedback, Double coding

## Abstract

**Background:**

This article outlines procedures for the feedback of individual research data to participants. This feedback framework was developed in the context of a personalized medicine research project in Canada. Researchers in this domain have an ethical obligation to return individual research results and/or material incidental findings that are clinically significant, valid and actionable to participants. Communication of individual research data must proceed in an ethical and efficient manner. Feedback involves three procedural steps: assessing the health relevance of a finding, re-identifying the affected participant, and communicating the finding. Re-identification requires researchers to break the code in place to protect participant identities. Coding systems replace personal identifiers with a numerical code. Double coding systems provide added privacy protection by separating research data from personal identifying data with a third “linkage” database. A trusted and independent intermediary, the “keyholder”, controls access to this linkage database.

**Discussion:**

Procedural guidelines for the return of individual research results and incidental findings are lacking. This article outlines a procedural framework for the three steps of feedback: assessment, re-identification, and communication. This framework clarifies the roles of the researcher, Research Ethics Board, and keyholder in the process. The framework also addresses challenges posed by coding systems. Breaking the code involves privacy risks and should only be carried out in clearly defined circumstances. Where a double coding system is used, the keyholder plays an important role in balancing the benefits of individual feedback with the privacy risks of re-identification.

**Summary:**

Feedback policies should explicitly outline procedures for the assessment of findings, and the re-identification and contact of participants. The responsibilities of researchers, the Research Ethics Board, and the keyholder must be clearly defined. We provide general guidelines for keyholders involved in feedback. We also recommend that Research Ethics Boards should not be directly involved in the assessment of individual findings. Hospitals should instead establish formal, interdisciplinary clinical advisory committees to help researchers determine whether or not an uncertain finding should be returned.

## Background

A feedback policy (also “communication” or “disclosure” policy) addresses a range of communications with participants such as re-contact to gather additional information or carry out new tests, dissemination of general study results, and the return of individual research data. Debate continues over what individual genetic data, if any, researchers should offer to participants [1]. Individual genetic data includes individual research results and incidental findings. Individual research results (“IRRs”) are results of the research project that concern the health status of individual participants. Incidental findings (“IFs”) also concern an individual participant’s health, but are findings encountered outside the objectives of the research project. Pressure to disclose these two forms of individual research data has mounted with the advent of new genetic sequencing technologies, which reveal swaths of health information, and an increasing emphasis on translational research. Susan Wolf [2] characterizes the question of feedback as “fundamentally a problem of translational science – a question of when information about an individual that is generated in research should be communicated for clinical attention”. A feedback policy should not only describe what kinds of individual research data will be returned to participants; it should also describe the procedures through which the data will be returned. But what form should feedback procedures take?

This article outlines an implementation framework for the return of individual research data (IRRs and IFs) to participants. It is based on our experience in developing a feedback policy for the Liver Disease Project (LDP) at the Research Institute of the McGill University Health Centre, the McGill University Health Centre and McGill University in Montréal, Québec. The LDP is a personalized medicine project mainly funded through a strategical grant awarded to the Research Institute by the *Fonds de recherche du Québec - Santé*. Our framework is suited to similar personalized research projects based in Canada, though it may offer a useful example to any research project communicating individual research data and using a coding system to protect participant privacy. This article does not aim to resolve the debate over *what* information, if any, should be returned to research participants. Feedback practices remain diverse across jurisdictions and research contexts and disagreement persists over whether or not researchers have an obligation to return IRRs and IFs. Instead, we focus on *how* information should be communicated to consenting participants once the decision to return IRRs or IFs has been made. The feedback process involves three central steps: assessing an IRR or IF, breaking the code to re-identify the participant, and communicating the finding to the participant.

This article is divided into a Background, Discussion, and Summary. The rest of this Background comprises of 3 subsections. The first subsection outlines the emerging ethical duty of researchers to return IRRs and IFs to participants. The second describes coding systems used to protect participant privacy. The third describes the LDP, along with its associated biobank, the Liver Disease Biobank, and details the Feedback Policy we developed for this project. In our Discussion, we identify procedural issues that may arise during the feedback of individual research data. First, “coding” of samples and data – delinking of personal identifiers from sensitive health information to protect privacy – complicates future communication and feedback to participants. Second, the roles of the principal investigator, the keyholder and the Research Ethics Board (REB) (the Canadian equivalent of an Institutional Review Board) in the feedback process are not adequately defined in the literature. We recommend that the REB’s role should be limited to the review and approval of the plan and procedures for the return of IRRs and IFs. Responsibility for assessing IRRs and IFs should remain with the principal investigator of the project. In uncertain cases, researchers should approach their clinical colleagues. Ideally, research institutions should establish a formal, interdisciplinary clinical advisory committee to assist researchers in this assessment. If a double coding system is employed, participants will need to be re-identified by a keyholder before they can be contacted. Re-identification involves privacy risks, and should only be carried out by the keyholder in clearly defined circumstances. We provide guidelines outlining the role and responsibilities of this keyholder. Procedural issues are serious obstacles to the ethical and efficient return of IRRs and IFs, yet they have received little attention in the literature and guidelines [3]. Our recommendations aim to provide direction for the return of IRRs and IFs, and to promote discussion about the roles of the actors involved.

### The researcher’s duty to return individual research results and incidental findings to participants

There is growing consensus in Canada that researchers have an ethical obligation to return certain health information to participants. This obligation is founded on the ethical principles of beneficence, reciprocity, and respect for persons. First, certain research findings could directly benefit individual participants. Second, communicating findings to participants gives them something in return for their contribution. Reciprocity reflects the idea “that individuals who voluntarily agree to contribute to research be provided some kind of access to the knowledge gained from such research” [4]. Finally, acting in the individual participant’s interest is a concrete expression of respect: acknowledgement that participants are more than simply a means to knowledge generation.

The literature distinguishes between four forms of participant feedback – general research results, baseline assessment results, IFs, and IRRs. These divisions may be far from clear in practice. The Public Partnership Project in Genomics and Society (P3G), an international consortium of population biobanks, offers the following definitions in a recent policy statement:

**Baseline Assessment**: includes measurements such as blood pressure, lung function, bone density, height, weight, fat, and others (taken at baseline or at any subsequent assessment).

**General Results**: aggregate results drawn from the analysis of data and samples of a group of research participants.

**Incidental Findings**: unforeseen findings concerning a research participant that have potential health or reproductive importance. They are discovered during the course of research but are outside its objectives.

**Individual Research Results**: results discovered during the course of research, which concern an individual participant, and have potential health or reproductive impact [5].

We focus on feedback procedures for IFs and IRRs from a biobank with a coding system. The return of individual research data raises distinct ethical and policy issues from the return of general results, and is more controversial than the return of baseline assessment results. To date, the debate has focused on determining what findings should be returned, either by defining general criteria, or by developing consensus-generating procedures in a given research community. Disagreement persists over what general substantive criteria must be met to trigger the obligation to return IFs or IRRs. This article does not aim to revisit this debate. It is, however, generally agreed upon in Canada that: “findings that are analytically valid, reveal an established and substantial risk of a serious health condition, and that are clinically actionable should generally be offered to consenting contributors” [6]. A list of major US and Canadian guidelines can be found in Table 1.

**Table 1:** **Research guidelines: return of IRRs and IFs to participants**

Network of Applied Genetic Medicine, Statement of Principles on the Return of Research Results and Incidental Findings (2013) (Summary) [7].	**Principle 2: Individual Results And Incidental Findings That Should Be Offered**
	Individual results and incidental findings should be offered to participants when:
	1) they are material, i.e. when the following conditions are met:
	1.1) they meet generally accepted criteria of scientific and clinical validity (criteria which are widely recognised by the medical community);
	1.2) they have clinical utility for the participant, i.e.:
	- the benefits associated with the communication of the results outweigh the risks;
	- prevention or treatment is available; and
	- individual, familial and social factors were considered;
	2) exceptions and additional considerations related to the research context have been weighed;
	3) REB approval has been obtained;
	4) the participant has consented to the return of results; and
	5) the research result has been confirmed.
	**Principle 3: Individual Results And Incidental Findings That May Be Offered**
	Individual results and incidental findings that are not compliant with the criteria set out in Principle 2 may be offered to participants (i.e. at the researcher’s discretion) when:
	1) they meet generally accepted criteria of scientific and clinical validity;
	2) the benefits of return surpasses the risks;
	3) REB approval has been obtained;
	4) the participant consented to the return; and
	5) the research result has been confirmed.
	**Principle 4: Individual Results And Incidental Findings That Have Implications For Family Members**
	It is possible that individual results and incidental findings have implications for the participant’s BIOLOGICAL RELATIVES. Under certain circumstances, these results may be returned (i.e. at the researcher’s discretion) to family members when:
	1) they meet the generally accepted criteria of scientific and clinical validity;
	2) the benefits of the return outweigh the risks;
	3) REB approval has been obtained;
	4) the research result has been confirmed;
	5) the participant agrees to share the result with biological relatives; and
	6) the biological relatives concerned agree to receive the results.
Public Population Project in Genomics and Society, Population Studies: Return of Research Results and Incidental Findings Policy Statement (2012) [5].	**Return of IRRs and IFs to Participants: Conditions and Modalities**
	*No Return of IRRs and IFs*
	There may be population studies where the policy is not to return individual results or findings, and this was consented to by participants at recruitment. This remains a viable option where appropriate. Researchers accessing the study population and their local Ethics Review Committee should be made aware of this policy.
	For population studies with a no-return policy or where participants did not consent at recruitment to the return of findings but have, nonetheless, consented to recontact for updates and for further questions or collection of samples, such a period can create an opportunity to explain and introduce a return of results and IFs policy and accompanying procedures, if the population study so chooses and with ethics approval. Indeed, upon recontact, participants could be provided with an option to consent (or not) to receiving such results. Moving forward, population studies with a no return policy could consider adding such an option to their consent process at recruitment.
	**Return of IRRs and IFs**
	*Decision to return results:* When consent to return results is present, one should consider whether the finding poses a material risk. Findings are material if they have:
	1) analytical validity;
	2) clinical significance; and
	3) actionability.
	Researchers, in collaboration with their local Ethics Review Committee, should consider returning IRRs and IFs to participants when they determine that the following criteria are met:
	1) the participant has consented thereto in the initial consent form or at a later time;
	2) the findings are analytically valid (ie, confirmed independently);
	3) they reveal a significant risk of a serious health condition; and,
	4) they are actionable.
	Researchers, in collaboration with their local Ethics Review Committee, may consider returning IRRs and IFs when the above criteria are not satisfied, but when the following criteria are met:
	1) the participant has consented thereto in the initial consent form or at a later time;
	2) the findings are analytically valid (ie, confirmed independently);
	3) they reveal an established risk of likely health importance to the participant; and
	3) they have a likely therapeutic benefit.
	The decision to return IRRs and IFs remains the responsibility of the researchers and the local Ethics Review Committee. Resources should be available for this decision-making process.
	*Communication of results.* Contacting participants for the communication of material findings remains the responsibility of the population study. The population study should ensure the quality of the results, as well as the timeliness and appropriateness of the information returned to a given participant (including considerations related to the number of recontacts).
	**Procedures**
	Population studies should put in place policies and procedures that clarify and circumscribe the obligations and procedures arising from their return of results policy. These should be reflected in any material-transfer agreements and access policies for researchers. These policies should include the length of duration of any return of results policy and the degree of involvement of researchers. Attention should be paid to issues of feasibility and reasonability. Procedures should be in place in the population study for the communication of such results by a health professional.
National Bioethics Advisory Commission, Research Involving Human Biological Materials: Ethical Issues and Policy Guidance, Vol. 1. (1999) [8].	Return results only if
	a. “the findings are scientifically valid and confirmed”
	b. “the findings have significant implications for the subjects’ health concerns” and
	c. “a course of action to ameliorate or treat these concerns is readily available.”
National Heart, Lung, and Blood Institute:	Recommendation 1: IRRs “*should* be offered to study participants in a timely manner if they meet *all* of the following criteria:
Fabsitz RR, McGuire A, Sharp RR, et al., Practical Guidelines for Reporting Genetic Research Results to Study Participants: Updated Guidelines from an NHLBI Working Group (2010) [9].	a. The genetic finding has important health implications for the participant and the associated risks are established and substantial.
	b. The genetic finding is actionable, that is, there are established therapeutic or preventive interventions or other available actions that have the potential to change the clinical course of the disease.
	c. The test is analytically valid and the disclosure plan complies with all applicable laws.
	d. During the informed consent process or subsequently, the study participant has opted to receive his/her individual genetic results.”
	Recommendation 4 “Investigators *may* choose to return individual genetic results to study participants if the criteria for an obligation to return results are not satisfied (see Recommendation 1) but all of the following apply:
	a. The investigator has concluded that the potential benefits of disclosure outweigh the risks from the participant’s perspective.
	b. The investigator’s Institutional Review Board (IRB) has approved the disclosure plan.
	c. The test is analytically valid and the disclosure plan complies with all applicable laws.
	d. During the informed consent process or subsequently, the study participant has opted to receive his/her individual genetic results.”
Canadian College of Medical Geneticists and the Canadian Association of Genetic Counsellors, Joint Statement on the Process of Informed Consent for Genetic Research (2008) [10].	**7(vii). Disclosure of results**
	Research participants should be informed at the outset if the results from the study will be disclosed and, if so, in what manner (e.g. individually to each participant or collectively as a study group through publication or another means). […] As indicated in section 7(v) above, researchers must take care to protect the privacy and confidentiality of individual participants in the course of results disclosure. Researchers should ensure that participants do not have unrealistic expectations with respect to disclosure of results. For example, a realistic estimate of the timeframe should be communicated to the participant. Ideally, participants should have the option to decline to be informed of study results at the time of enrollment or at any time during the study. It is recommended that for all studies in which results will be disclosed, genetic counselling should be a component of the informed consent process. The counselling provided should be appropriate to the clinical impact of the study. It should be provided at a level of depth and by staff with a level of training and expertise that is appropriate for the complexity of the information being explained.
	Clinically Significant Results: It is recommended that any clinically-significant laboratory results ascertained through a research laboratory and disclosed to the research participant be validated in an accredited clinical diagnostic laboratory to ensure that appropriate quality assurance measures have been followed. Accredited clinical diagnostic laboratories that offer confirmation of research findings can be identified using online databases such as GeneTests.org.
	Unexpected Results: Genetic research is unique in that there is the potential to obtain information about individuals or families that was unanticipated. In addition, it is possible that in the course of studying one disease, a researcher may discover that an individual, family or community is at increased risk for another, possibly unrelated, disorder. If individual results are to be disclosed, research participants should be made aware of the possibility that unexpected results could be obtained and should be informed of policy with regards to disclosure of such results in the context of significant health implications for the individual and/or his family. Prior consent should be obtained with regard to the research participant’s wish to be informed of these unanticipated results
Wolf SM, Lawrenz FP, Nelson CA, et al. Managing Incidental Findings in Human Subjects Research: Analysis and Recommendations (2008) [11].	Researcher *should* disclose IFs likely to offer strong net benefit from participant’s perspective: (a) genetic information revealing significant risk of a condition likely to be life-threatening; (b) genetic information that can be used to avoid or ameliorate a condition likely to be grave; and (c) genetic information that can be used in reproductive decision-making: (1) to avoid significant risk for offspring of a condition likely to be life-threatening or grave or (2) to ameliorate a condition likely to be life-threatening or grave.
	Researcher *may* disclose IFs offering possible net benefit from participant’s perspective: (a) genetic information revealing significant risk of a condition likely to be grave or serious, when that risk cannot be modified but a research participant is likely to deem that information important; and (b) genetic information that is likely to be deemed important by a research participant and can be used in reproductive decision-making: (1) to avoid significant risk for offspring of a condition likely to be serious or (2) to ameliorate a condition likely to be serious.
	Researcher *should not* disclose IFs offering unlikely net benefit from the participant’s perspective, including information whose likely health or reproductive importance cannot be ascertained.
Zawati HZ et al. Reporting Results from Whole-genome and Whole-exome Sequencing in Clinical Practice: a Proposal for Canada? [12] (endorsed by the Canadian College of Medical Geneticists)	Prior to using [Whole Genome or Whole Exome Sequencing], physicians need to explain to their patients the nature of the results that could arise, so as to allow them to make informed choices over whether to take the test and which results they wish to receive.
	Results revealing a clinically significant condition that is actionable during childhood should be reported to the parents. Parents cannot refuse to receive such results.
	The child’s views should be solicited and given due weight and consideration in accordance with his or her age and maturity.

The extent of feedback of IRRs or IFs to participants will depend on the research context. The research protocol determines what information is collected and how it is analyzed, and therefore determines the likelihood that the research will generate findings of clinical relevance to participants. Other aspects of the research context determining the scope of the researcher’s duty to return individual research data are the vulnerability and dependence of the study population; whether a clinical relationship already exists between researcher and participant; the intensity and duration of their interactions; and the availability of adequate funding, personnel, and technology. (For a complete list, see [3], Table One; [13]). Extensive variation exists between jurisdictions, research projects, and the types of information they will return to participants. Often, researchers opt to return only limited subsets of IRRs or IFs. They may decide to return only IRRs, only IFs, both or neither. In some cases, it may be difficult to distinguish these two categories of research data. The framework we present is relevant to researchers planning to return any type of individual research data, and is particularly suited to the personalized medicine research context, described below.

Quite apart from the debate over what individual feedback to offer participants, are procedural questions of how to assess individual research data, re-identify participants, and communicate sensitive health information to them. These questions are largely overlooked in the literature. This oversight is understandable for several reasons. Uncertainty about what information should be returned overshadows the question of *how* it will be returned. The heterogeneity of research context and biobanks inhibits the generation of policy guidance for return procedures [3]. Uneven treatment of substance and procedure is evident in existing guidelines. For example, major Canadian research funding bodies stipulate that IFs should be disclosed if “interpreted as having significant welfare implications for the participant” [14]; the definition given for “welfare” is extremely broad. However, where such findings are likely, researchers are simply instructed to “develop a plan indicating how they will disclose such findings to participants” [14]. No guidance is provided as to what such a plan might need to include.

The problem of ambiguity and inconsistency identified in policies at the national level becomes acute when research relies on biobanks, especially where the biobank is intended to serve researchers residing in different jurisdictions, each having its own policy on the matter. The problem is further compounded by limited awareness on the part of researchers and funding organizations of the costs of designing and implementing procedures for the feedback of individual health data. The factors determining these costs must be contemplated by “researchers in planning their budgets and funding agencies in determining the appropriate level and duration of funding” [15]. In addition to costs, risks must also be considered. Offering personal feedback of health information may promote the therapeutic misconception and induce participation. The return of uncertain information may also upset participants, or lead them to undergo unnecessary and potentially harmful follow-up testing.

### The relationship between privacy protection and feedback of individual data

A central complication for the feedback of IFs or IRRs is the coding of biomaterials and data. Many research projects rely on biobanks that code their materials to protect participant privacy and prevent unauthorized access to participant health information. The risk of unauthorized identification of participants is an important characteristic of data intensive health research projects. It influences the extent of ethics review and oversight that is required, if any. In some countries it may also determine how privacy laws apply to the storage, use, and dissemination of research samples and data. Privacy laws generally apply to “identifying information” and “identifiable individuals” [16]. A common approach in research is to distinguish between direct identifiers (name, health card number, telephone number etc.) that alone or in combination “obviously” identify an individual; and quasi-identifiers (postal code, date of birth etc.) that may allow indirect re-identification. The danger is that a third party outside the therapeutic or health research relationship, such as an employer or insurer, links sensitive health information (such as a genetic predisposition to a disease) to a specific individual. This risk, however, is not altogether easy to define or mitigate. Health research generally relies on two privacy protection mechanisms: anonymization or coding [17]. Although inconsistencies remain in the terminology used by stakeholders, anonymization is generally defined as the irreversible removal of personal identifiers and quasi-identifiers from sensitive health data. This mechanism is becoming increasingly unpopular, especially in genetics research, as it greatly reduces the scientific value of the samples over time [17]. A more promising alternative is coding: replacing identifiers with a unique, random code. The identifiers are stored in a separate database along with the code so that the process can be reversed. Security can be further intensified with “double coding”, where “the codes associated with the original data and the identifier data set are different, and the information linking them is kept in a separate linking database” [16]. This linking database is administered by an independent third party intermediary, or “keyholder”. This double coding structure is explained in more detail in the following section.

Where data is coded, researchers must link individual health data back to personal identifiers in order to contact the participant. Indeed, in studies involving the irrevocable stripping of personal identifiers (“anonymization”), the return of IRRs or IFs is rendered virtually impossible [6, 18]. In summary, research projects contemplating feedback of IRRs or IFs will have to consider procedures for both re-identification and communication.

### The Liver Disease Project

**Figure 1:**
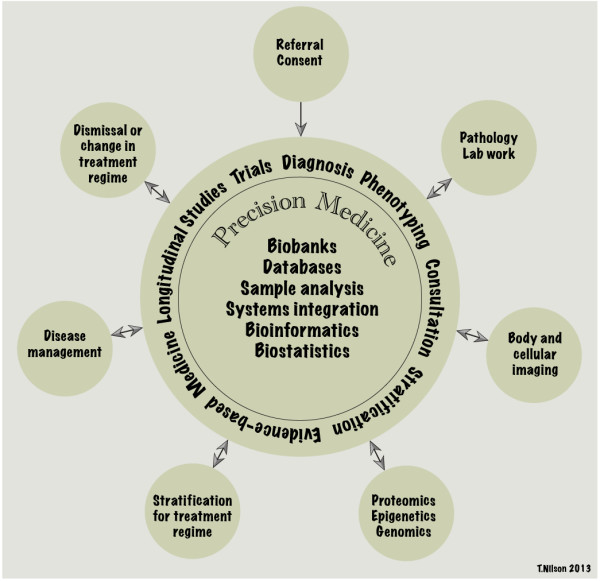
**The precision medicine approach of the Liver Disease Project.** Legend: (None). *This is an original figure.

The proposed framework addressing assessment, re-identification, and communication was inspired by our efforts to develop a feedback policy for the Liver Disease Project (LDP) located at Research Institute of the McGill University Health Centre in Montréal, Québec. The LDP integrates clinical management of liver disease with high-end research analysis to identify new biomarkers for the diagnosis, staging, and eventual treatment of liver disease. Its main focus is complex polygenic and chronic liver disease, namely Non-Alcoholic Fatty Liver Disease (NAFLD), and its progression to Non-Alcoholic Steatohepatitis (NASH), which puts patients at risk of developing terminal liver disease. NAFLD comprises a spectrum of liver conditions associated with the accumulation of triglycerides in liver hepatocytes in the form of lipid droplets, inflammation and fibrosis. The LDP embraces a “personalized” or “precision” medicine approach, generating vast amounts of clinical data on patients to identify biomarkers related to disease progression and response to treatment. (See Figure 1) Knowledge of these biomarkers could lead to improvement in individual prognoses and development of targeted treatments [19]. As discussed above, this patient-centric research context heightens the intensity of the researchers’ duty to feedback individual research data. In other areas of research, such as population biobanking, this feedback may be much more difficult to implement [5]. Our framework is most suitable for personalized medicine research projects in Canada. It may provide a useful example to researchers in other jurisdictions and research contexts contemplating the feedback of individual research data.

The liver samples used for analysis by LDP researchers, along with detailed, associated clinical information, are stored in a Liver Disease Biobank (“Biobank”). Funded by the *Fonds de recherche du Québec – Santé*, the Biobank is a collection of high quality liver specimens taken mostly from patients during surgical intervention, where tissue is removed as part of treatment. To protect these sensitive health data and samples, the Biobank relies on a double coding system. Double coding is the separation of personal identifiers and samples (along with associated clinical or research data) into two separate databases. For each patient, one numerical code is assigned to the personally identifying information, and a second, distinct code is assigned to the samples and data. These two databases are mediated by a third “linkage” database that contains a list pairing the two numerical codes for each patient. Access to this third database is necessary for re-identification of patients. For added security, the linkage database is separately maintained, and accessible only by an independent physician (the “keyholder”). In short, the LDP is part of a double coded “biobank research system” [6], comprising surgical collection of samples, storage in the Liver Disease Biobank, and access by LDP researchers for analysis. As we discuss below, double coding protections present difficulties for the handling and communication of IRRs and IFs.

The LDP applies general assessment criteria to identify IRRs and IFs. These general criteria are recommended by several policy guidelines (See Table 1):

- Did the participant **consent** to return?
- Is the finding **analytically valid**? Analytical validity considers the capacity of a test to measure the characteristic it is designed to identify. In particular, is the test precise, reliable, accurate, positive if the genetic characteristic is present (analytical sensitivity), and negative if it is absent (analytical specificity)?
- Is the finding **clinically valid**? Clinical validity is a measure of the accuracy with which a test identifies or predicts a clinical condition. It is defined in terms of diagnostic specificity and sensitivity, positive predictive value, and negative predictive value.
- Is the finding clinically useful, i.e. actionable? **Clinical utility** considers the value of the results in guiding the patient’s choices regarding prevention or therapeutic strategies. The assessment of the utility requires consideration of both the benefits and the potential risks associated with the knowledge of the result or indicated intervention.

In the Biobank consent form, participants were asked to express their preferences regarding feedback of individual health data:

“If research using your samples reveals information we were not looking for (i.e. incidental findings) and which clearly indicate a significant health problem that can be treated or prevented, then you will be informed by providing us a name of a physician who will transmit the information to you.”

“I would like to be informed, through my physician, about incidental findings that indicate a significant health problem. (**YES/NO**)”

Note that participants are given the option of designating a physician to mediate the return of any significant health information. Now that we have presented the context, and the types of information we want to return to participants, we turn to a discussion of the difficulties encountered in the handling and communication of individual research data to participants.

## Discussion

A host of procedural issues hinder feedback of individual research data [20]. Both assessment and communication of IRRs and IFs require re-identification – breaking of the double coding system – which generates privacy risks. A feedback policy must carefully specify when, and in what circumstances, a participant should be re-identified. The roles of the Research Ethics Board, the linkage database keyholder and the “designated physician” (the clinician designated by the participant in the consent form to mediate the communication of sensitive individual health information) remain unclear. We outline these roles, and recommend that hospitals establish a formal, interdisciplinary advisory committee to help researchers review uncertain findings. Feedback policies must also contemplate cases where participants refuse to be contacted, and provide mechanisms to keep participant contact information and feedback preferences up to date.

### Re-identification

Three procedural steps are required to return individual research data: researchers must assess the analytical validity, clinical validity (together “scientific validity”) and clinical utility of an IRR or IF; the affected participant must be re-identified; and the participant (or a designated physician) must be contacted [6]. Researchers who commit to return IRRs of IFs must ensure adequate resources and expertise are available to carry out these steps. Determining the order of the steps, however, is deceptively straightforward. Because breaking the code creates privacy risks, it has been assumed that scientific validities and clinical utility should be confirmed before re-identification. We argue that in some cases it is preferable to re-identify the participant *before* such confirmations.

Confirming scientific validity involves expensive tests and expert assessments. If participants can opt to refuse re-contact about IRRs or IFs (as is the case with the LDP), this preference should be established before confirming scientific validity. It is wasteful to confirm validity only to discover upon re-identification and inspection of the consent form that the participant has refused re-contact. Québec’s Network of Applied Genetic Medicine (one of the few organizations offering procedural guidance on the return of IRRs and IFs) recognizes this problem, and suggests that scientific validity be established after re-identification: “[i]n order to minimize the operational impact of this condition on clinical laboratories, confirmation of the [genetic] results should only be performed when the result satisfies [the other conditions]” [7] (See Table 1).

Re-identification may also be necessary to fully assess clinical utility. Unlike scientific validity, clinical utility may require consideration of personal characteristics. Assessing clinical utility involves weighing the benefits and risks of disclosing individual health information. Disclosure, however, will have little utility if the health risk has already materialized, or if the participant has passed away. Beyond these obvious examples, many guidelines employ a broad definition of utility including subjective factors such as participants’ reproductive interests, or whether the information has implications for their family members [6, 12, 21]. The definition may also include personal utility for life planning, encompassing return of information on predispositions to untreatable late-onset conditions. A biobank’s feedback policy should clearly indicate how clinical utility will be assessed, and whether or not re-identification should precede such assessment. It should always be kept in mind that re-identification carries risks to privacy and confidentiality of data. Linkage should not be carried out without a good reason.

Assessing whether to return a finding before re-identification may be even more problematic if a popular “binning” approach is used for the return of IFs. Binning is the grouping of IFs into broad categories, usually along the lines of scientific validity and clinical utility. Each category can receive a different treatment by researchers, such as “must return”, “may return”, “do not return.” For “may return” findings, participants are asked whether or not they want to receive the information. This process allows participants to express their informational preferences in a streamlined fashion [22]. Offering a comprehensive set of options for the return or refusal of information, however, may increase the need to re-identify before confirming other conditions.

With foresight, researchers can address these conundrums. For example, participant preferences about re-contact could be stored along with their research data and sample, or even integrated into the numerical codes during de-linkage. For example, a 0 added to the end of a participant code could signify a refusal to be re-contacted. Adding a 1 could signify a preference for return. This way, re-identification would not be required in order to assess the consent criterion. Consideration of personal characteristics may also be precluded before re-identification, or even before re-contact. To address this problem, assessment criteria must be carefully selected and ordered to balance the privacy risk of re-identification against the cost of assessment. For example, it may not be worthwhile to require researchers relying on a coded biobank to consider subjective factors in the analysis of clinical utility, (e.g. impact on reproductive decision making), if such factors will remain unknown before re-identification. Alternatively, objective aspects of clinical utility could be considered before re-identification, and subjective aspects could be considered afterwards.

### The role of the Research Ethics Board

What role should the Research Ethics Board (REB) play in handling IRRs and IFs? We argue that the REB should not develop the feedback plan or assess whether or not an IRR or IF should be returned. It should be researchers who develop the plan, assess and confirm findings, and communicate with participants and their designated physician. If the researchers analyzing the data have limited expertise, or are analyzing limited data sets, this should be adequately reflected in the original feedback plan and consent form. The primary role of the REB should be to prospectively review the researcher’s feedback policy. The researcher would be responsible for implementation. Such an approach is endorsed by Québec’s Network of Applied Genetic Medicine’s 2013 *Statement of Principles on the Return of Research Results and Incidental Findings*.

Apart from the *Statement*, there is little explicit description of the REB’s role in the handling of IRRs and IFs in other guidelines. Canada’s *Tri-Council Policy Statement: Ethical Conduct for Research Involving Humans* (2010) (“TCPS2”) does not currently describe procedures for handling IRRs and IFs, except to say that researchers should “develop a plan” of how they will communicate to participants and submit to the REB (art 3.4). This rule applies to all genetic researchers (art 13.2(a)) and other researchers where material IFs are likely. The steward of the policy, the Interagency Advisory Panel on Research Ethics, is proposing to amend the TCPS2 to provide an expanded role for REBs in handling procedures. The proposed text would read: if material IFs are discovered, “researchers shall report them to the REB … The researcher should provide enough information to enable the REB to determine whether the [IFs] are material, and to assess the risks and benefits of disclosing the findings to the participant” [23]. If these proposed changes are accepted, REBs would become responsible for confirmation of IRRs and IFs.

**Figure 2:**
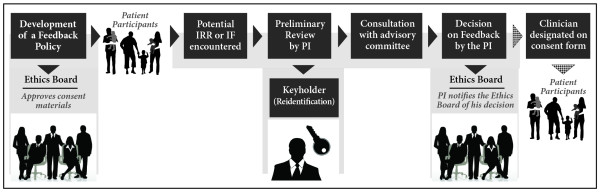
**Outline of a feedback procedure for IRRs and IFs.** Legend: This image details the steps in a procedure for the feedback of individual research data, and the roles of the Research Ethics Board (REB), Keyholder, and Principle Investigator (PI). *This is an original figure.

REBs should not have the responsibility to assess the risks and benefits of returning individual IRRs or IFs encountered in research. They are unlikely to have the clinical and scientific expertise to make this type of assessment for research projects. Assessments also require significant time, expense, and resources already in short supply for REBs. Researchers, on the other hand, are more likely to have the expertise and familiarity with their own scientific methods to assess IRRs and (albeit to a lesser extent) IFs, or to know clinicians that do. If researchers feel compelled to deviate from a previously approved return plan, or are uncertain about a particular complex finding, they can consult their clinical colleagues or their REB on a case-by-case basis. Ideally, a formal, clinical advisory committee would be established and maintained in research hospitals. It would convene on an ad-hoc basis at the request of principal investigators to evaluate findings. Given the issues involved in deciding to return IRRs or IFs, this committee would include physicians, geneticists, ethicists, and lawyers. Our recommendation is supported by recently published U.S. guidelines: “Researchers who discover an unanticipatable incidental finding of concern should assess its significance, consulting with experts as appropriate” [24]. This flexible approach allows for the rapid return of clear and serious health risk information, while providing for deliberation in less certain cases. Once a decision is taken to return findings, the principal investigator should communicate directly with the participant or designated physician. Principal investigators should not seek approval from the REB before communication; rather, they should notify the REB of the decision and the means of communication. The complete return procedure we propose is illustrated in Figure 2.

### Keyholder guidelines

In a biobank with a double coding system, the linkage database that contains the numerical codes linking samples and data to personal identifiers is held by a trusted intermediary or “keyholder.” A researcher wishing to feedback individual research data must first re-identify the affected participant. Re-identification implicates the keyholder in the feedback procedure. We provide guidelines for the keyholder’s role here:

1. The linkage database must be kept separate from the databases holding personal identifiers and sensitive health information.
2. To the extent possible, the keyholder should be independent from the research project.
3. The keyholder should be a physician informed of the criteria for return and able to assess them. To adequately safeguard participant privacy, the keyholder should be instructed of all situations where re-identification is necessary, and the conditions related to each.
4. The keyholder is authorized to re-identify participants in certain circumstances, which should be clearly delimited at the start of the project.
5. Before re-identification for the purpose of feeding back IRRs or IFs to individual participants, the keyholder must receive a request in writing from the biobank director. This written request will confirm that the participant has consented to return, and that the return criteria (analytical/clinical validity etc.) have been met.
6. The keyholder will take all reasonable measures to ensure the participant’s privacy before and after linkage.
7. The keyholder will keep a written record of all re-identifications and requests for re-identification.

The role and responsibilities of the keyholder must be clearly articulated at the start of a research project to ensure that the double coding system does not prevent return of important health information to participants, or unnecessarily put their privacy at risk.

### Additional procedural issues

Replacing personal identifiers with a code, or a double code, reduces the number of identity cross-checks and increases the chance of an error in sample labelling and tracking [3]. Biobanks planning to provide personal feedback will want to ensure the utmost care in sample tracking and labelling. Return of erroneous risk information to participants may lead to unnecessary anxiety and follow-up, and may even expose researchers to legal liability if harm results.

Researchers may offer participants the option to refuse re-contact about health information. This respects their right not to know, their right to “decide whether or not to be informed of the results of genetic information and the resulting consequences…” [25]. An individual’s interest in not knowing may be heightened in the research context, as they have not expressly sought out diagnosis. Some suggest that the option to refuse re-contact in some research contexts should extend even to treatable, clinically significant findings [26]. The fidelity of such refusals, however, may be suspect, especially if a serious risk is encountered years later, or if the participant’s opinion or attitude towards research changes. In biobanking projects, researchers may have little or no contact with participants after the original consent. It is difficult to know if participants’ wishes expressed in the original consent form continue to reflect their true wishes. Refusal to be re-contacted tends to be given broadly: individuals refuse to be re-contacted about a general category (or categories) of findings. Researchers may therefore suspect that participants opting-out of return of individual research data did not fully understand what they were refusing. In addition, researchers confronted by serious, valid, and actionable IRRs or IFs may feel that their professional duty to warn (or rescue) the participant (and the correlative risk of legal liability for failure to do so) may over-ride the participant’s refusal to be informed [13]. As a compromise, some authors have suggested that researchers should “reconfirm with a research participant who indicated refusal that he or she is electing to decline information” on all IRRs or IFs [11]. The opposite problem could also occur: a researcher may attempt to return a result to a participant who opted for re-contact, only to find that the participant is no longer interested. The Network of Applied Genetic Medicine recognizes this latter situation, and recommends that researchers “confirm the desire to receive results before returning them” [7]. But how can this desire be ascertained? Perhaps the initial communication to the participant can be broad and impersonal in order to conceal risk information (as much as practically possible) until their preferences are established. But this could delay treatment or cause participants to misapprehend the nature of the risk researchers hope to communicate. Alternatively, direct communication to participants may undermine their control over the flow of information. This is a classic difficulty with respecting the right not to know: it may be impossible to divine the participant’s preference without revealing something about the nature of the risk information.

Participant contact information and re-contact preferences should be updated where possible, and procedures should be in place to address situations where the affected participant is not readily contactable. Our Feedback Policy for the LDP requires the principal investigator to take reasonable but limited steps to locate a participant, such as asking the participant’s treating physician, asking known relatives, or carrying out a basic internet search. The final communication also poses procedural challenges and requires design choices. Some researchers suggest that disclosure be made “directly to the research participant” [11]. Others prefer disclosure to the research participant’s primary care physician [27]. Disclosure directly to a physician, however, may deprive the participant of control over his or her health information and privacy [11]. The participant should be able to decide who to consult and what is entered into his or her medical records. Another solution, adopted by our project, is to ask the participant to designate a physician on the consent form to mediate the return of significant individual health information. Feedback policies should also be careful to clearly limit the scope of the duties of researchers (including temporal limitations). For Wolf and colleagues [6], “the goal [of return is] not to supply clinical care or a clinical work-up of the finding, but to put the participant in the position to make an informed decision about what next steps to take, including seeking clinical work-up and care.” Finally, both the participant and/or the designated physician may need to be informed of the possibility of false positives due to an inaccurate test result (in the case of an IF) or a coding error during re-identification. Where feasible, follow-up testing should be recommended.

## Summary

The return of IRRs and IFs is an essential frontier for the translation of research findings into clinical solutions. Clear, efficient and ethical processes are needed to guide the handling of IRRs and IFs, and ultimately to improve patient care. Our central recommendations are summarized here:

-  Researchers planning to return IRRs and IFs should prospectively develop a clear procedure for the review and communication of findings. This procedure should take into account the privacy protections in place and clearly articulate the role of researchers, the REB, and the keyholder in the return process.
-  The primary role of the REB is to prospectively review the researcher’s return policy and procedures. The REB should not generally be directly involved in the review of candidate IRRs and IFs, but should be notified of the advisory committee’s recommendation and the principal investigator’s decision on whether or not to communicate findings.
-  A formal, interdisciplinary clinical advisory committee should be established, preferably by research institutions, to convene on an ad-hoc basis and advise researchers on the handling of uncertain findings.
-  If a double coding system is employed, the role of the keyholder in the return process needs to be clearly articulated. The keyholder should be informed of the situations where IRRs and IFs will be returned, and the conditions that must be met to justify re-identification.
-  Researchers should facilitate the update of participant contact information and provide participants with the opportunity to express clear preferences about re-contact.

Coding protection systems are important means of privacy protection, but present a hurdle to the feedback of individual research data to participants. Researchers can avoid some issues by upstream design. Projects should outline clear procedures for the assessment of individual research data, re-identification of participants, and communication to participants and designated physicians. The role of the REB in the handling of IRRs and IFs should generally be limited to review of the researcher’s feedback policy. The REB may, however, act as an advisor when researchers are uncertain of how to proceed, especially if an advisory committee has not been established. REBs should also be notified of the principal investigator’s feedback decisions. Researchers relying on biobanks using double coded systems will also need to provide clear guidance for the keyholder responsible for the linkage database. Finally, researchers and their funders must take into account the considerable costs and infrastructure required for the design and implementation of a feedback policy.

## Abbreviations

IF: Incidental finding
IRR: Individual research result
LDP: Liver disease project
REB: Research ethics board.
